# Supplementary material for: FcαRI co-stimulation converts human intestinal CD103+ dendritic cells into pro-inflammatory cells through glycolytic reprogramming
Source: Nat Commun. 2018 Feb 28;9:863. doi: 10.1038/s41467-018-03318-5 (PMC5830413; doi:10.1038/s41467-018-03318-5)
Supplement: Supplementary file 1 — Supplementary Information [file 41467_2018_3318_MOESM1_ESM.pdf]

Supplementary information

FcαRI co-stimulation converts human intestinal CD103$^+$ dendritic cells into pro-inflammatory cells through glycolytic reprogramming

Hansen et al.

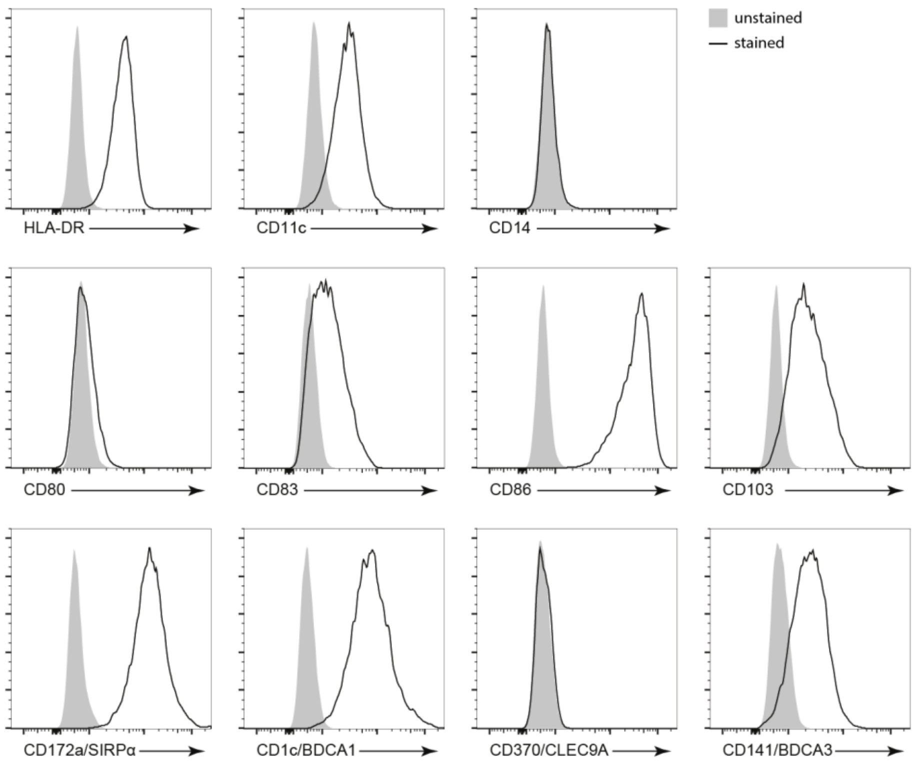

Supplementary Figure 1. Phenotypical analysis of *in vitro* generated CD103$^+$ DCs.

Expression analysis of various dendritic cell (DC) markers and identifying markers for intestinal subsets on unstimulated *in vitro* generated CD103$^+$ DCs. Light grey histogram indicates background staining. Representative example of three experiments using different donors.

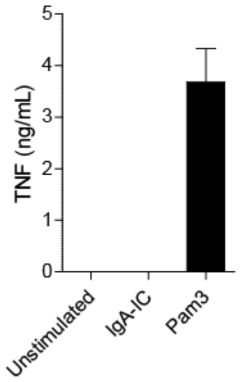

Supplementary Figure 2. IgA-IC endotoxin levels are too low to affect cytokine production.
Monocyte derived dendritic cells (moDC) were stimulated with IgA immune complexes (IgA-IC) or Pam3CSK4 (Pam3). Experiments were performed in triplicate. After 24h cytokine levels were analysed using ELISA, mean + SEM. Representative example of five experiments using different donors.

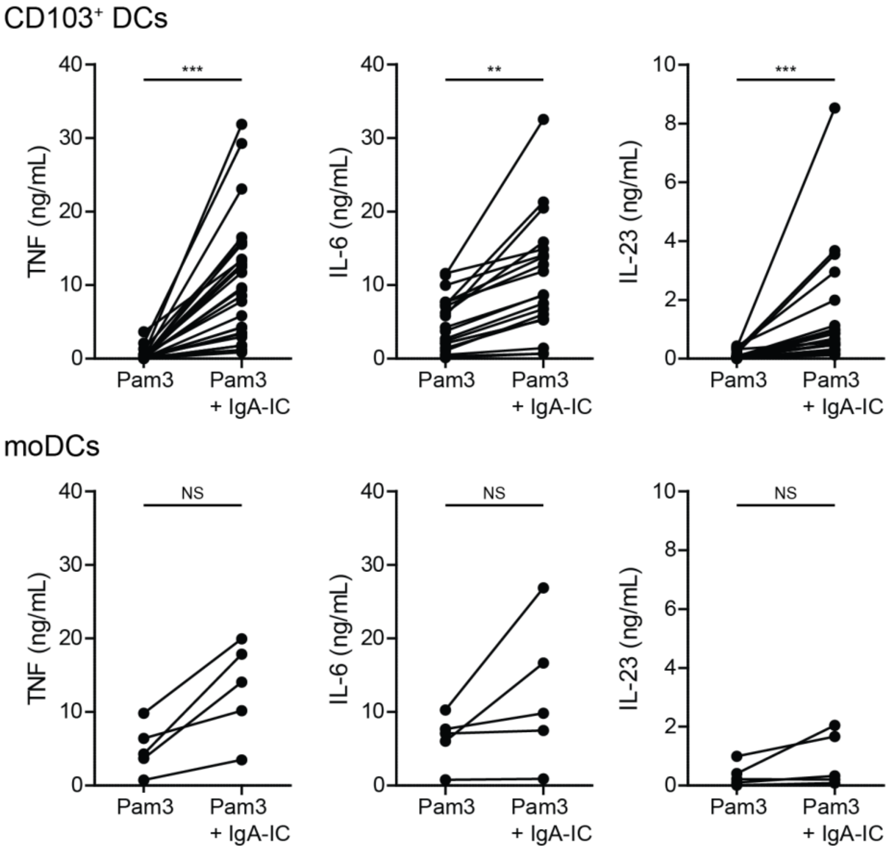

Supplementary Figure 3. IgA-IC promote pro-inflammatory cytokine production by CD103$^+$ DCs, but not by moDCs.

CD103$^+$ dendritic cells (DC) and moDCs were stimulated with Pam3CSK4 (Pam3) or Pam3CSK4 combined with IgA immune complexes (IgA-IC). Each pair of dots represents one donor. *p <0.05, **p <0.01, ***p <0.001, NS not significant, Mann Whitney test.

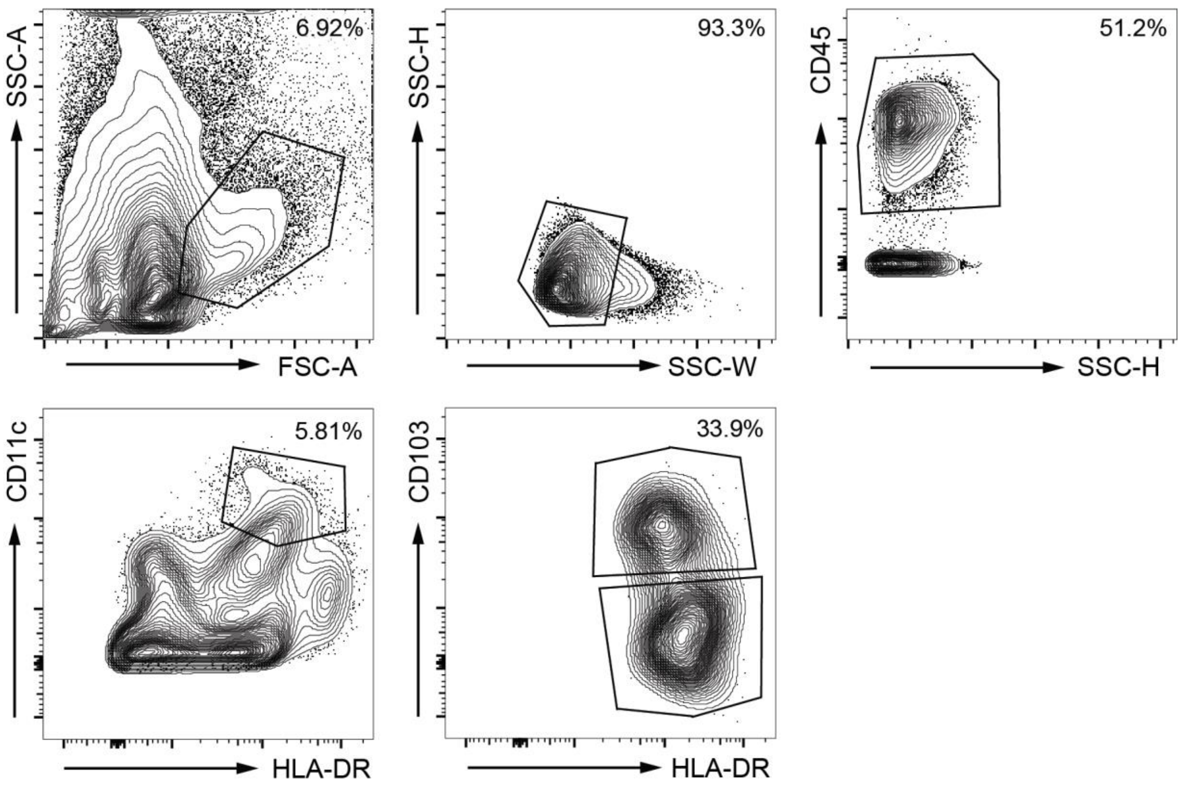

Supplementary Figure 4. Cell sorting strategy of primary CD103^{+} dendritic cells.

Gating strategy of cell sorting of CD103^{+} dendritic cells (DC) from surgically removed intestine. After single cell selection, CD45^{+}CD11c^{+}HLA-DR^{+}CD103^{+} or CD45^{+}CD11c^{+}HLA-DR^{+}CD103^{-} cells were sorted and used for analysis in Figure 2D (CD103^{+}) and Supplementary Fig. 5 (CD103^{-}), 8 (both), and 14 (both). One representative sample of seven donors.

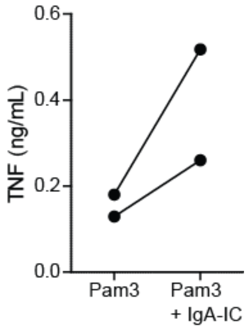

Supplementary Figure 5. CD103⁻ DCs increase TNF production upon FcαRI co-stimulation.
TNF protein production by CD103⁻ dendritic cells (DC) isolated from human colon after stimulation with Pam3CSK4 (Pam3) or Pam3CSK4 combined with IgA immune complexes (IgA-IC). Each pair of dots represents one donor.

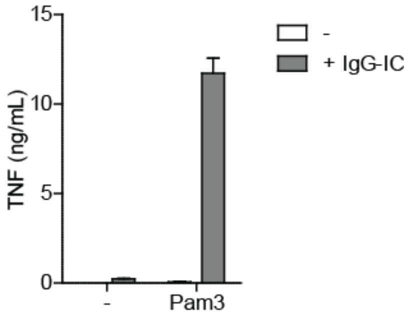

Supplementary Figure 6. IgG-IC promote pro-inflammatory cytokine production in combination with TLR stimulation.

$CD103^+$ dendritic cells (DC) were stimulated with Pam3CSK4 (Pam3), IgG immune complexes (IgG-IC) or a combination. Experiments were performed in triplicate. After 24h cytokine levels were analysed using ELISA, mean + SEM. Representative example of four experiments using different donors.

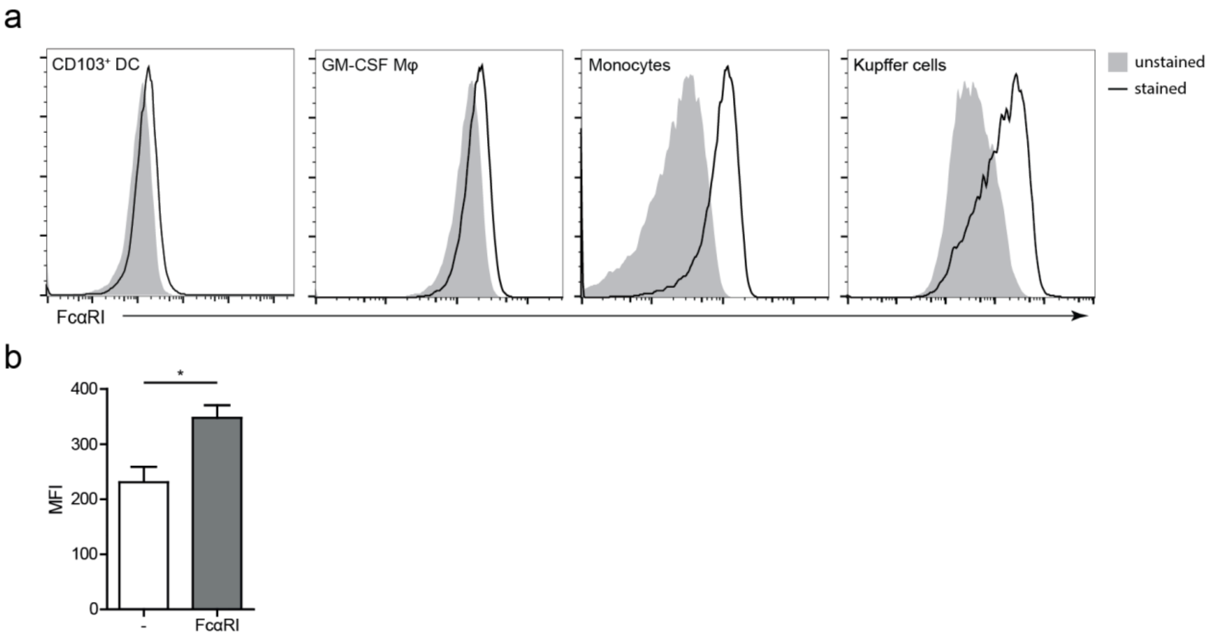

Supplementary Figure 7. Comparison of FcαRI expression by different immune cells.

A CD103^{+} dendritic cells (DC) and GM-CSF differentiated macrophages (Mφ) were analysed for FcαRI expression using flow cytometry. Light grey histogram indicates background staining. Monocytes and Kupffer cells were measured as a positive reference. Representative example of three experiments using different donors. **B** Mean fluorescent intensity (MFI) of CD103^{+} DCs stained for FcαRI. Pooled data of three different donors, mean + SEM. *p <0.05. Student's *t* test.

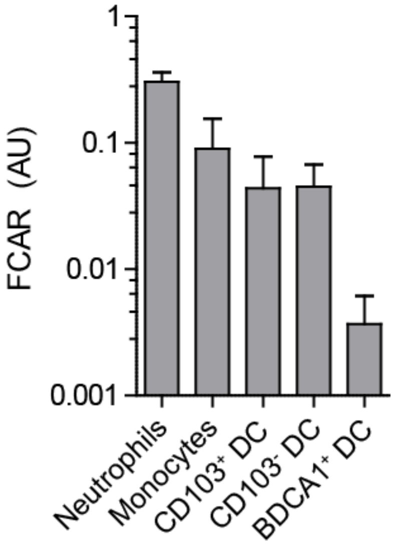

Supplementary Figure 8. *FCAR* expression in primary CD103$^+$ dendritic cells compared to other primary immune cells.

mRNA expression of FcαRI encoding gene *FCAR* (normalized to *GAPDH*) in neutrophils, monocytes, and BDCA1$^+$ dendritic cells (DC) from blood and CD103$^+$ or CD103$^-$ DCs isolated from human colon. Pooled data from three different donors for each cell type, mean + SEM.

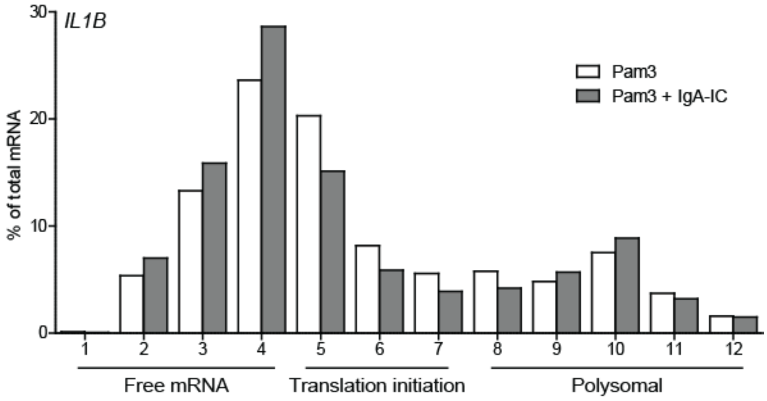

Supplementary Figure 9. FcαRI-TLR cross-talk does not affect *IL1B* translation.

Lysates of CD103^{+} dendritic cells (DC) stimulated for three hours with Pam3CSK4 (Pam3) or Pam3CSK4 with IgA immune complexes (IgA-IC) were loaded on sucrose gradients to measure mRNA translation of *IL1B* (normalized to *GAPDH*). Representative example of three experiments using different donors.

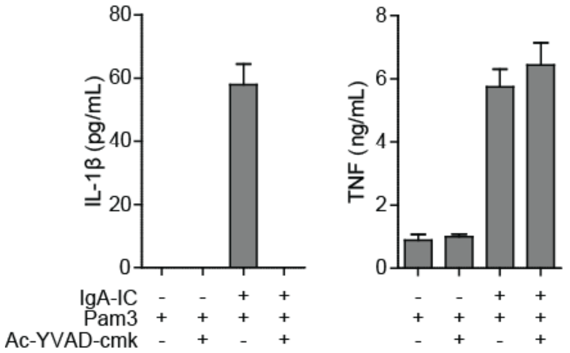

Supplementary Figure 10. Activation of caspase-1 through FcαRI triggering is essential for IL-1β production.

Cytokine production by CD103$^+$ dendritic cells (DC) stimulated with Pam3CSK4 (Pam3), IgA immune complexes (IgA-IC) or a combination after treatment with 20 µM Caspase-1 inhibitor Ac-YVAD-cmk. Experiments were performed in triplicate. After 24h cytokine levels were analysed using ELISA, mean + SEM. Representative example of two experiments.

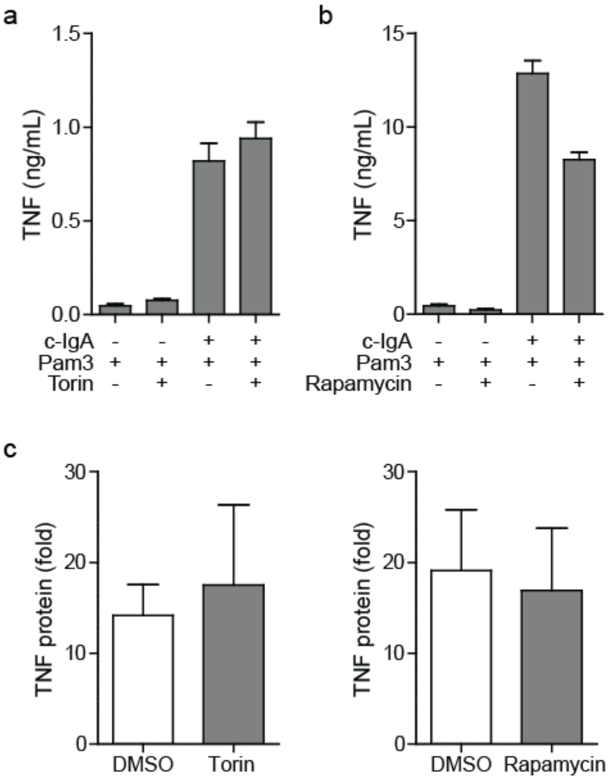

Supplementary Figure 11. IgA-IC-TLR ligand co-stimulation is not dependent on mTOR.
Cytokine production by CD103^{+} dendritic cells (DC) stimulated with Pam3CSK4 (Pam3), IgA immune complexes (IgA-IC) or a combination after treatment with 100 nM mTORC1/mTORC2 inhibitor Torin (**A**) and 20 nM mTORC1 inhibitor Rapamycin (**B**). Experiments were performed in triplicate. After 24h cytokine levels were analysed using ELISA, mean + SEM. Representative example of at least three different experiments. **C** CD103^{+} DCs were stimulated with Pam3CSK4 or Pam3CSK4 combined with IgA-IC in the presence of DMSO, rapamycin, or Torin. Data shown is protein production normalized to Pam3CSK4-induced TNF production for each experiment, mean + SEM of five (rapamycin) and three (Torin) experiments.

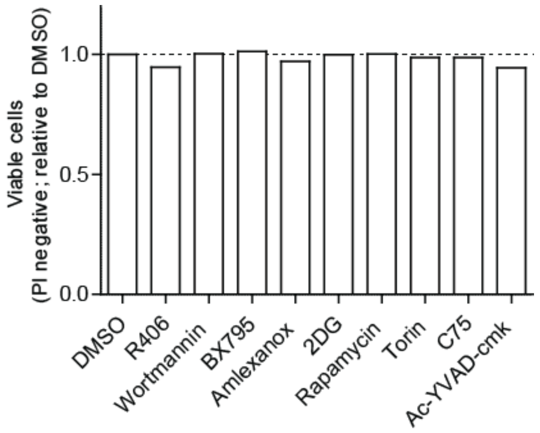

Supplementary Figure 12. Inhibitor treatment does not affect cell viability.

CD103$^+$ dendritic cells (DC) were treated overnight with indicated inhibitors or DMSO. Cells were analysed using flow cytometry for viability by propidium iodide (PI) staining. Data shown is viability staining normalized to DMSO-treated cells.

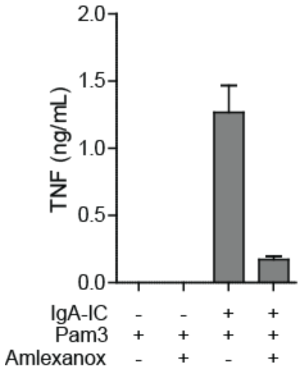

Supplementary Figure 13. IgA-IC-TLR ligand co-stimulation is dependent on TBK1-IKKε.
Cytokine production by $CD103^{+}$ dendritic cells (DC) stimulated with Pam3CSK4 (Pam3), IgA immune complexes (IgA-IC), or a combination after treatment with 100 μM TBK1-IKKε inhibitor Amlexanox. Experiments were performed in triplicate. After 24h cytokine levels were analysed using ELISA, mean + SEM. Representative example of two experiments using different donors.

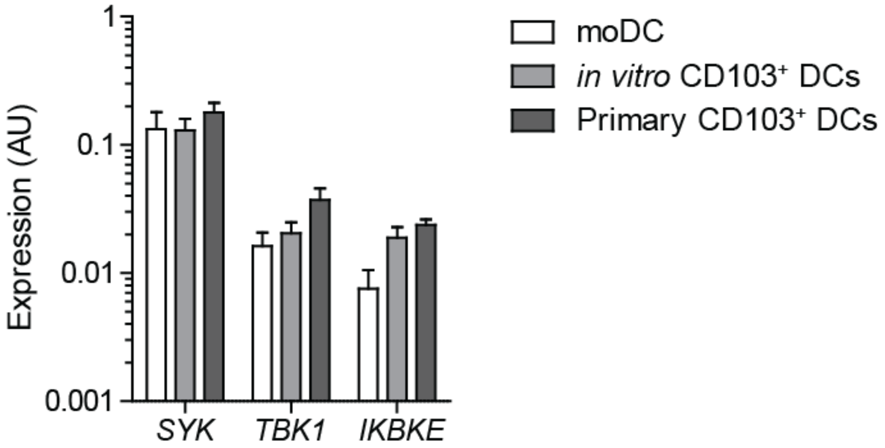

Supplementary Figure 14. Expression analysis of signalling molecules in primary CD103$^+$ DCs. Expression of *SYK*, *TBK1*, and *IKBKE* (relative to *GAPDH*) in primary CD103$^+$ dendritic cells (DC) was analysed using qPCR and compared to *in vitro* generated CD103$^+$ DCs and moDCs (the latter two cell types are positive controls, since they are known to express these molecules). Pooled data of three different donors, mean + SEM.
